# Temperature sensitivity patterns of carbon and nitrogen processes in decomposition of boreal organic soils – Quantification in different compounds and molecule sizes based on a multifactorial experiment

**DOI:** 10.1371/journal.pone.0223446

**Published:** 2019-10-10

**Authors:** Ari Laurén, Mari Lappalainen, Antti-Jussi Kieloaho, Kristiina Karhu, Marjo Palviainen

**Affiliations:** 1 School of Forest Sciences, Faculty of Science and Forestry, University of Eastern Finland, Joensuu, Finland; 2 Natural Resources Institute Finland (Luke), Helsinki, Finland; 3 Department of Forest Sciences, University of Helsinki, Helsinki, Finland; Centro de Edafologia y Biologia Aplicada del Segura, SPAIN

## Abstract

Climate warming and organic matter decomposition are connected in a recursive manner; this recursion can be described by temperature sensitivity. We conducted a multifactorial laboratory experiment to quantify the temperature sensitivity of organic carbon (C) and nitrogen (N) decomposition processes of common boreal organic soils. We incubated 36 mor and 36 slightly decomposed *Carex-Sphagnum* peat samples in a constant moisture and ambient temperature for 6 months. The experiment included three temperature and two moisture levels and two food web manipulations (samples with and without fungivore enchytraeid worms). We determined the release of carbon dioxide (CO_2_) and dissolved organic carbon (DOC) in seven molecular size classes together with ammonium N and dissolved organic N in low molecular weight and high molecular weight fractions. The temperature sensitivity function Q_10_ was fit to the data. The C and N release rate was almost an order of magnitude higher in mor than in peat. Soil fauna increased the temperature sensitivity of C release. Soil fauna played a key role in N release; when fauna was absent in peat, the N release was ceased. The wide range of the studied C and N compounds and treatments (68 Q_10_ datasets) allowed us to recognize five different temperature sensitivity patterns. The most common pattern (37 out of 68) was a positive upwards temperature response, which was observed for CO_2_ and DOC release. A negative downward pattern was observed for extractable organic nitrogen and microbial C. Sixteen temperature sensitivity patterns represented a mixed type, where the Q_10_function was not applicable, as this does not allow changing the sign storage change rate with increasing or decreasing temperature. The mixed pattern was typically connected to intermediate decomposition products, where input and output fluxes with different temperature sensitivities may simultaneously change the storage. Mixed type was typical for N processes. Our results provide useful parameterization for ecosystem models that describe the feedback loop between climate warming, organic matter decomposition, and productivity of N-limited vegetation.

## Introduction

Climate warming has been greatest in high latitudes [1] where boreal forests are located. Boreal forests and peatlands are significant global carbon (C) reservoirs; upland forest soils contain 90–500 Pg C and peatlands 260–600 Pg C [2]. Decomposition of soil organic matter continuously returns a share of the stored C back to the atmosphere. Therefore, decomposition has a key role in global greenhouse gas (GHG) balance. Northern forests and peatlands tend to accumulate soil C because of a slow decomposition rate that is limited by low nitrogen (N) content, cool temperature, soil acidity, and scarce decomposer communities [3,4]. Decomposition is a complex process where organic matter quality, physical conditions such as prevailing temperature, moisture and oxygen (O_2_) supply, N availability, and soil microbes and fauna are closely linked and affect the rate of decomposition and the quality of the released substances. Depending on these interactions, a different proportion of carbon dioxide (CO_2_) and dissolved organic carbon (DOC) in various molecule sizes is released [5].

Decomposition and climate are connected in a recursive manner; CO_2_ emission from organic matter decomposition enhances global warming, which again stimulates the rate of decomposition. This recursion can be described by temperature sensitivity. Understanding the global GHG balance requires quantification of the feedback loops between decomposition, primary production, N availability, and temperature [6]. As the rate of primary production in N-limited systems depends largely on the N supply from the decomposing organic material, the temperature sensitivity of N release in decomposition is equally important to net C exchange and ultimately to the global GHG balance as is the temperature response of CO_2_ efflux *per se*.

Temperature sensitivity is typically quantified by the Q_10_ value, which describes the relative change in the C or N release rate under a 10-degree change in temperature (e.g. [7]). Although Q_10_ values for CO_2_ release have been studied rigorously, the temperature sensitivity of N processes is still poorly understood [8,9,10]. According to the carbon quality-temperature hypothesis (CQT) presented by Davidson and Janssen [11], Q_10_ is related to the chemical composition of decomposing material and several environmental constraints. This can be seen as an increase in Q_10_ value with decreasing site fertility [7] and as higher Q_10_ values for subsoil than for topsoil [12]. Beier et al. [13] (2008) found that Q_10_ can be higher in cool climates, making Northern ecosystems particularly vulnerable to climate warming [14]. While Q_10_ for CO_2_ emissions is rather well established, remarkably less is known about the temperature sensitivity of DOC release; studies addressing the release of DOC in different molecule sizes have not appeared until recently [15].

The tight coupling of C and N processes is an essential feature in the GHG feedback loop, as primary production and growth of soil microbes strongly depend on N availability [9,16]. In decomposition, C and N cycles can have an asymmetrical temperature response, where C dynamics follow the exponential Q_10_ curve, but N can be insensitive to temperature change and responds to moisture changes or other environmental constraints [13,17,18]. On the other hand, Weedon et al. [14] demonstrated a very temperature sensitive response of N availability and productivity in northern peatland and highlighted the role of soil microbial function in regulating temperature sensitivity.

There is a close functional connection between soil microbes and soil fauna. Whereas soil microbes are the primary decomposers, soil fauna play an important role in C and N release by grazing on soil microbes and consumption of detritus [19,20,21]. In boreal soils, enchytraeid worms such as *Cognettia sphagnetorum* are functionally the most important faunal group [22,23,24,25]. Enchytraeid worms can double the CO_2_ and DOC release from temperate peat soil [26] and significantly enhance N, CO_2_, and DOC release from boreal organic soils [5,27,28]. The temperature sensitivity of worm activity is largely unknown; however, it is likely that the function of soil fauna responds to temperature change and is therefore indirectly linked with GHG balance. Although the importance of the interactions between soil fauna, microbes, and decomposition in GHG balance has been recognized [26,29], building these interactions into simulation models requires further studies to expose the hidden interactions between C and N processes. Model development will benefit especially from multifactorial decomposition experiments [18] that differentiate the effect of soil type, temperature, moisture, and soil food webs. Such studies are currently absent in the body of scientific literature.

The aim of this study was to quantify the temperature sensitivity of C and N release in boreal organic soils using a controlled laboratory experiment. We incubated the most common boreal organic soils, mor from upland forest sites and *Carex-Sphagnum* peat from drained forested peatland, and studied the release rate of CO_2_, high-molecular weight (HMW) DOC, low-molecular weight (LMW) DOC, DOC in different relative molecule size classes, NH_4_-N, HMW-DON, and LMW-DON during the experiment. Incubation was conducted at +5°C, +10°C, and +20°C and in two moisture levels. Furthermore, we incubated soil with the presence and absence of enchytraeid worms. Temperature sensitivity patterns were evaluated for each soil type and soil faunal treatment. This study continues the works presented by Laurén et al. [27] and Lappalainen et al. [5,28], who demonstrated the role of soil fauna and soil type on different C and N release rates but did not study the effect of abiotic factors, such as temperature and moisture on C and N release.

## Material and methods

### Study setup

**Fig 1:**
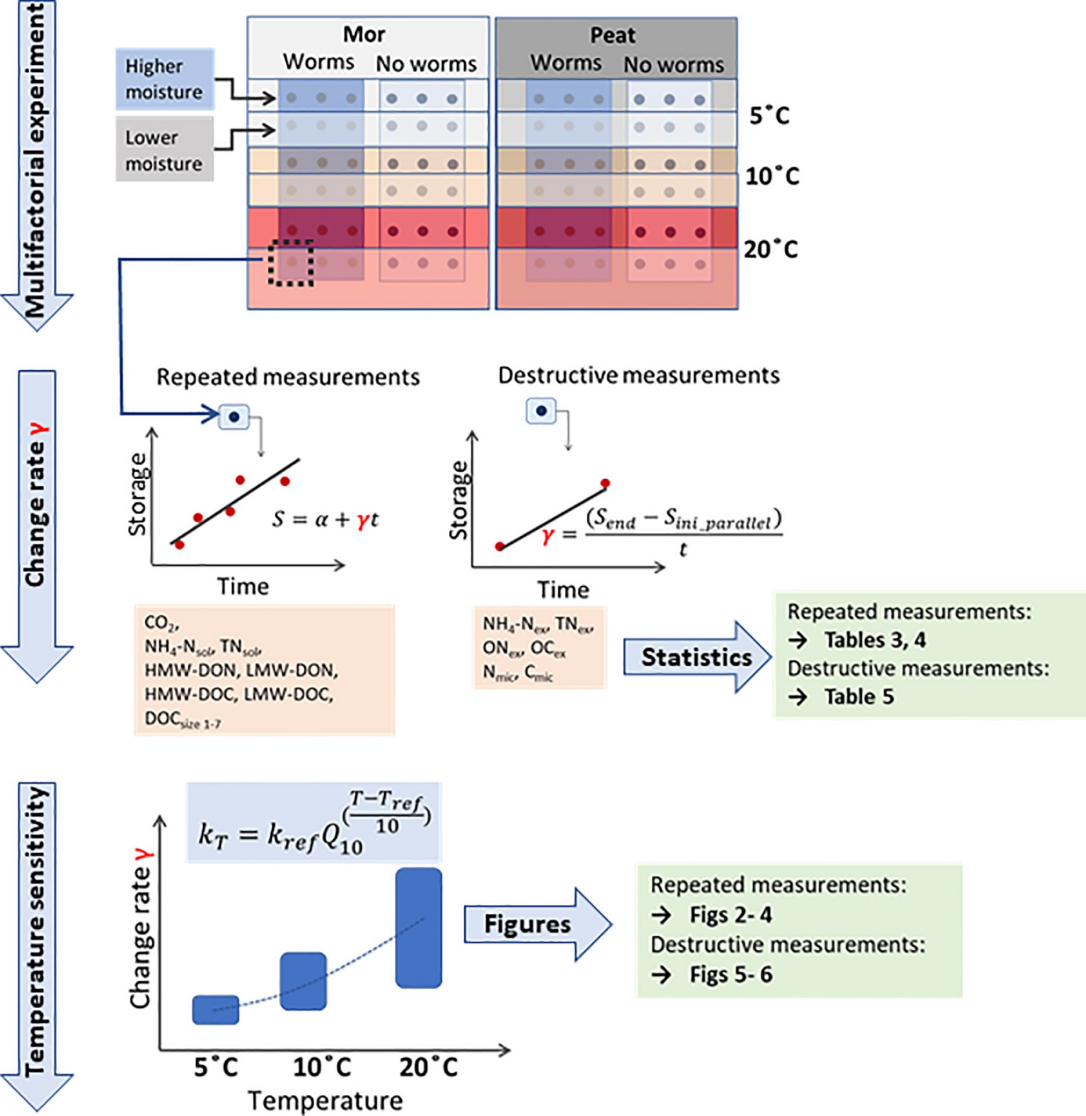
Study flowchart. In the **multifactorial experiment** we incubated two soil types in three different temperatures at two moisture levels and in the presence or absence of soil fauna. Three replicates were included in each treatment. The incubation time was 6 months. **Repeated measurements** were conducted during the experiment; CO_2_ efflux was measured from the top of the sample and storage of soluble C and N were determined at different molecular sizes (NH_4_-N_sol_, TN_sol_, DOC, and DON in high-molecular-weight (HMW) and low-molecular-weight (LMW) fractions, and DOC in size classes 1–7). **Rate of storage change** (γ, μg g^-1^ day^-1^) was determined as the slope of the time-storage data of the repeated measurements. Analyses requiring **destructive measurements** (extractable and microbial C and N, denoted with subscripts “ex” and “mic”) were conducted after the experiment. The change in these storages was evaluated with respect to the initial storage of parallel samples. The change rate (γ, μg g^-1^ day^-1^) was determined by dividing the observed change with the duration of incubation. The effect of the experimental factors on the observed change rates were tested using linear mixed effect models (Tables 2–4). **Temperature sensitivity** analysis was conducted using the observed change rates (γ). Higher and lower moisture levels were combined (n = 6 for γ) and Q_10_ function was fit to the data. The observed temperature sensitivity patterns and the Q_10_ values are presented in Figs 2–6.

We conducted a multifactorial laboratory experiment to study the temperature sensitivity of C and N release in decomposition of boreal organic soils. The study flowchart is presented in Fig 1. We studied storage change rates of 13 different C compounds and 11 N compounds; the abbreviations and descriptions of the studied compounds are summarized in Table 1.

**Table 1:** Abbreviations and description of the measured compounds in the multifactorial experiment.

Variable	Description	Method
**Repeated measurements**		
CO_2_-C	Carbon dioxide efflux	Static chamber, IR analyzer
DOC	Dissolved organic carbon	TOC-500A
HMW-DOC	High-molecular-weight DOC in soil solution, >1 kDa	Ultrafiltration, TOC-500A
LMW-DOC	Low-molecular-weight DOC in soil solution, <1kDa	Ultrafiltration, TOC-500A
DOC_size1_	Largest relative molecule size of DOC	HPLC
DOC_size2_—DOC_size2_	Relative molecule size of DOC in descending order	HPLC
DOC_size7_	Smallest relative molecule size of DOC	HPLC
NH_4_-N_sol_	Ammonium nitrogen in soil solution	FIA-Star 5020
NO_3_-N_sol_	Nitrate nitrogen in soil solution	FIA-Star 5020
TN_sol_	Total nitrogen in soil solution	FIA-Star 5020
DON	Dissolved organic nitrogen	FIA-Star 5020
HMW-DON	High-molecular-weight DON in soil solution, >1 kDa	Ultrafiltration, FIA-Star 5020
LMW-DON	Low-molecular-weight DON in soil solution, <1kDa	Ultrafiltration, FIA-Star 5020
**Destructive measurements**		
OC_ex_	Organic C, extracted with K_2_SO_4_	TOC-5000A
C_mic_	Microbial C	Fumigation-extraction
NH_4_-N_ex_	Ammonium nitrogen, extracted with K_2_SO_4_	FIA-Star 5020
NO_3_-N_ex_	Nitrate nitrogen, extracted with K_2_SO_4_	FIA-Star 5020
TN_ex_	Total nitrogen, extracted with K_2_SO_4_	FIA-Star 5020
ON_ex_	Organic N, extracted with K_2_SO_4_	FIA-Star 5020
N_mic_	Microbial N	Fumigation-extraction

### Study sites and soil sampling

The boreal landscape is characterized by a mosaic of upland forests and open or forested peatlands [30]. For this study, soil samples were collected from the middle boreal region in Sotkamo, eastern Finland, from sites that represent the most common upland and peatland site types in Finland [31,32,33], namely mesic upland forest and drained forested peatland with *Carex-Sphagnum* peat and medium fertility. The long-term (1981–2010) mean annual precipitation in the area was 591 mm, with about 40% falling as snow; the mean annual air temperature was +2.3°C [34]. The upland site (Kangasvaara 63°51’N /28°58’E, altitude 220 m a.s.l.,) is dominated by old-growth Norway spruce (*Picea abies* (L.) Karsten) with Scots pine (*Pinus sylvestris* L.) mixture and has been described in detail by Finér et al. [35]. The mor layer thickness varied from 5 to 9 cm. The peatland site (Koivupuro 63°52’N /28°39’E, altitude 200 m a.s.l.) with Scots pine stand developed on peat layer with a depth of 1–5 m and with a slightly decomposed peat (H3-H4 on the von Post [36] scale of decomposition) in the top 40 cm of the profile. The Koivupuro site has been described earlier by Ahtiainen and Huttunen [37] and Haahti et al. [38].

The sampling was conducted along two parallel lines (15 m apart) along which the soil samples were collected. The microsite for the sampling was a topographically even spot located at least 1 m away from the nearest tree. A cylindrical core (diameter 20 cm, height 9–15 cm) was cut from the organic soil layer to fit into a plastic container (diameter 20 cm, height 20 cm). The mean dry mass for the incubated soil cores was 253 g for mor and 279 g for peat. All living above-ground vegetation was carefully removed from all samples. The sampling procedure followed that of Lappalainen et al. [5].

The experiment included two soil types (S_m_ = mor, S_p_ = peat), three temperature levels (T_5_ = +5°C, T_10_ = +10°C, and T_20_ = +20°C), two moisture levels (θ_1_ = lower moisture, θ_2_ = higher moisture), and two food web manipulations (W_0_ = without enchytraeid worms, W_1_ = with worms) with three laboratory replicates in each treatment. Therefore, the total number of samples was 72. For moist soil samples, one plastic container represented one replicate. To ensure an adequate supply of soil solution, two containers of lower-moisture mor represented one replicate. During the incubation the same volume of soil solution from both containers was collected for chemical analyses. In addition, four parallel soil samples for each soil type were collected for analyses of extractable C and N contents and the other basic soil characteristics requiring a destructive sampling. Additional mor material was also collected for extraction of the enchytraeid worms that represent the soil faunal function in the experiment. Enchytraeids were inoculated into half of the soil containers as described below. The soil was stored at +4°C prior to the worm extraction.

### Preceding soil analyses and preparation for incubation

All samples were defaunated by exposing them to a freeze-thaw cycle that was repeated twice. The freeze-thaw cycle may have caused a momentary C and N flush, which was considered in the calculation by omitting the first measurement and thereafter considering the long-term rate of C and N release. Laurén et al. [27] and Lappalainen et al. [5] described the sample pre-treatment and defaunation and discussed the consequences of the defaunation on soil microbes and fauna.

The moisture level in the samples was adjusted using the following procedure: gravitational water was first allowed to drain through a hole in the bottom of the containers for all samples. Soil samples representing lower moisture conditions (treatment θ_1_) were then allowed to dry through evaporation at +4°C. In peat, the drying was further enhanced using suction samplers (described below). For higher moisture treatment (θ_2_), the mor samples were irrigated with deionized water whereas the peat samples were kept in the initial water content. We were able to generate a modest moisture difference between θ_1_ and θ_2_ treatments (Table 2), where the higher moisture level corresponds to approximately -3 kPa and the lower to -8 kPa matric potential [39,40]. Extending the moisture difference towards drier conditions would have limited the use of suction samplers in the incubation. In the present setup, the collection of the soil solution sample took 3 days in the θ_1_ treatment for mor; the time tends to increase considerably towards drier conditions. A time domain reflectometer (TDR) was used to monitor the initial moisture. The water content during the experiment was kept constant by adding water into the container until the initial mass was achieved after soil solution sampling.

**Table 2:** Basic characteristics of soil used in the incubation.

	Mor	(sd)	Peat	(sd)
C/N	33.5	1.7	40.5	0.9
soil pH (in H_2_O)	3.97	0.01	3.51	0.06
water content vol-%				
θ_1_	35	6	63	6
θ_2_	40	5	71	8
Loss on ignition, %	86	6	98	0.3
Bulk density, kg m^-3^	81	9	74	6
Enchytraeids ^a^				
T_5_	22	4	9	4
T_10_	71	11	12	5
T_20_	>100 ^b^		32	9
Total C μg g^-1^ om^c^	599419	10636	524235	μ
Total N μg g^-1^ om^c^	17907	678	12959	300
OC_ex_	8686	1931	5208	832
C_mic_	7 342	3641	6641	1947
DOC	1301	345	674	190
LMW-DOC	415	128	230	57
ON_ex_	581	75	248	22
NH_4_-N_ex_	94	21	24	2
N_mic_	832	137	291	89
NH_4_-N_sol_	84	36	44	15
DON	36	38	17	12
LMW-DON	0.02	0.05	0.03	0.03
NO_3_-N_sol_	0.04	0.05	0.06	0.02

^a^ 1000 worms m^-2^, calculated as means from the analyzed layers.

^b^ Results for T_20_ in mor is an estimation due to fragmentation of the worms that made the calculation unreliable.

^c^ om denotes organic matter.

For the incubation, the containers were placed in a dark growth chamber (GR77, Conviron Controlled Environments Ltd.) with constant ambient temperature (+5°C, +10°C, and +20°C) and relative humidity of 80%. Half of the samples were inoculated with enchytraeids, which were extracted from the additional mor material using a wet funnel method [41]. At least 50 worms per container were inoculated at the beginning of the incubation and a further 50 worms were inoculated monthly to ensure the continuous presence of enchytraeid population in the containers. The number of enchytraeids inoculated during the experiment (per container) corresponds to a total of approximately 8 000 individuals m^-2^, or 0.1 g m^-2^ in worm dry mass. The total number of enchytraeids in the soil containers was determined at the end of the incubation.

At the beginning of the experiment, the soil CN ratio, pH, and contents of extractable C and N compounds were determined by means of parallel soil samples (Table 2). The total C and N content in the soils was determined using a CHN analyzer (LECO CHN-2000). The soil pH was measured from a suspension of soil in H_2_O (1:2 v:v). C and N compounds were extracted by 0.5 M K_2_SO_4_ solution and the extracts were filtered through a 0.45-μm filter. Total extractable N (TN_ex_), extractable NH_4_-N (NH_4_-N_ex_), and extractable NO_3_-N (NO_3_-N_ex_) were analyzed by flow injection analysis (FIA-Star 5020 Analyzer, FOSS TECATOR). Extractable organic C (OC_ex_) was analyzed by a total organic carbon analyzer (TOC 5000A, Shimadzu). The extractable organic N (ON_ex_) content was calculated by subtracting NH_4_-N_ex_ and NO_3_-N_ex_ from TN_ex_. Extractable compounds are assumed to include both the compounds in the solution and the compounds adsorbed on solid surfaces. The microbial biomass C (C_mic_) and N (N_mic_) were determined using the fumigation-extraction method [42, 43, 44].

### Repeated measurements during the incubation

Repeated measurements included monitoring of CO_2_ efflux and C and N storage in the soil solution (Fig 1, Table 1). Six measurement events were conducted with 3- to 7-week intervals during the incubation period of 179 to 185 days. The slight difference in the incubation time results from the time-consuming sampling and measuring sequence and the large number of containers in the experiment. CO_2_ efflux from the soil containers was measured using a static chamber method with an infrared gas analyzer (ADC LCA-2, the ADC Bioscientific Ltd.) just before each soil solution sampling. The equipment and the procedure have been described in detail by Lappalainen et al. [5].

Soil solution was trapped from the containers using suction samplers (see [5] for procedural details). Three sampling tips (MacroRhizon with syringe, Eijkelkamp, length 9 cm, diameter 4.5 mm) were inserted into the soil and approximately 35 to 100 ml of soil solution was extracted in each sampling event.

The extracted soil solution was divided into two subsamples, one for determining C and N content and the other for measuring pH. The first subsample was further divided into two parts; one was ultrafiltered through a membrane with a nominal molecular weight limit of 1 kDa (Amicon Stirred Cell model 8400, pressure 1.5–2 bar) and one remained unfiltered. Prior to the ultrafiltration, the solution was diluted with 2 M KCl (sample:KCl of 3:1) to prevent flocculation and retention of the molecules on the membrane. In the filtration, two thirds of the load volume were allowed to pass through the membrane. The ultrafiltered fraction represents the low molecular weight fraction of the DOM.

The total dissolved N (TN_sol_), NH_4_-N_sol_, NO_3_-N_sol_, and DOC were determined from both the ultrafiltered and the unfiltered samples (TOC-5000A, FIA-Star 5000 Analyzer). DON content was calculated as the difference between TN_sol_ and mineral N (NH_4_-N_sol_ + NO_3_-N_sol_). Although NO_3_-N_sol_ was included in the calculations, it is not presented in the figures and tables because the amount was marginal throughout the experiment. The relative molecular-size distributions of DOC were determined from soil solution samples by using size-exclusion chromatography analysis (HPLC, Agilent Technologies, USA) as described in Lappalainen et al. (2018). Seven different relative molecular-size classes were distinguished on the basis of the peaks in the chromatography results. Size classes 1 and 2 represented high-molecular-weight DOC (HMW-DOC), i.e. > 1 kDa, and classes 3–7, LMW-DOC. Ultrafiltration and HPLC were used in parallel as together they provide a more precise picture of the molecular-size distribution in soil solution; HPLC provides the relative molecular-size distribution where the actual molecular size remains unknown, whereas the compounds passed through the ultrafiltration membrane are known to be < 1 kDa.

### Destructive analyses at the end of incubation

At the end of the experiment, the volume and the mass of the soil in the containers were measured and the soil was cut vertically into four similar sectors. The soil volume was calculated from the height of the soil core and the diameter of the cylinder-shaped container. The mass of the first sector was determined before and after drying at 105°C to estimate the volumetric water content. The dried sector was then further used to determine the loss on ignition (LOI) at 550°C. The second sector was cut horizontally into 5-cm slices, from where enchytraeids were extracted using the wet funnel method. The third sector was used for analyses of OC_ex_, TN_ex_, N_mic_, and C_mic_. For lower moisture treatment, where two containers formed together one replicate, the mean over the containers represented the soil characteristics for the replicate.

### Data processing and statistical analyses

We selected incubation time such that the change in C and N storage would be linear in time. The studies by Conant et al. [45] and Hamdi et al. [46] suggest that C release rate at the beginning of incubation is highest but stabilizes after the labile C has been consumed (approximately after 50 days). Our previous experiments [5,27,28] with the same soil types show that the compound storage in the repeated measurements change linearly between 50 to 180 days of incubation. Linearity allowed us to use a single value, the rate of storage change (γ), as a response variable in the further analyses, thus facilitating a balanced comparison of different treatments and compounds. Due to linearity, the slight difference in the incubation time is not likely to affect the temperature sensitivity results.

The repeated measurements were processed to derive the rate of storage change in the following manner: for soil solution samples we kept record for the quantity of the substances that were removed in the soil solution extraction and considered that in the further data processing. We first added the removed quantities to the substance storage dataset and then standardized the datasets by dividing them with the dry mass of the organic fraction of the sample. We next omitted the first sampling occasion (time < 50 days) to avoid the effect of the C and N flush and to linearize the data. The original dataset is available as S1 Dataset. We then fitted a linear mixed effect model (Eq 1) to the remaining storage-time dataset. All statistical analyses were performed using the lme package in R version 3.5.3. The rate of change (μg g^-1^ day^-1^) of C and N fractions was expressed as the slope (*γ*_*ijkm*_) of the line where the x-coordinate represents elapsed time and the y-coordinate represents storage [5,27]. To make the CO_2_ release data directly comparable to the soil solution data, we constructed a cumulative release dataset by assuming that the observed CO_2_ release continues with constant rate until the next measurement. The CO_2_ dataset was thereafter treated similarly to the soil solution data. The application of the linear mixed effect model allows statistical comparison of the treatment effects and their interactions.

$$Q_{ijkmnp}=\alpha_{ijkm}+\gamma_{ijkm}t_{ijkmnp}+a_{ijkmn}+c_{ijkmn}t_{ijkmnp}+e_{ijkmnp}$$ (1)

where *Q*_*ijkmn*_ is the measured compound content per mass unit of soil (μg g^-1^ dry mass) in the soil type i (S_m_, S_p_), moisture level j (θ_1_, θ_2_), temperature k (T_5_, T_10_, T_20_), and worm treatment m (W_0_, W_1_), replicate n, and sampling event p. In the fixed part of the model, α is the intercept term for soil type i, moisture level j, temperature k, and worm treatment m. The intercept term includes worm-temperature, worm-moisture, worm-soil type, temperature-moisture, temperature-soil type, and moisture-soil type interaction terms. *γ*_*ijkm*_ represents the release rate of the compound (μg g^-1^ d^-1^) and *t*_*ijkmnp*_ is the time (d, days) from the beginning of the experiment. The release rate term includes the interactions as the intercept term does. The random component includes the intercept *a*_*ijkm*_, the slope *c*_*ijkmn*_, and the residual term *e*_*ijkmnp*_.

The rate of change for the storages requiring destructive sampling (ON_ex_, NH_4_-N_ex_, OC_ex_, N_mic_, C_mic_) was calculated as the difference between the end stage and a mean storage measured from parallel samples at the beginning of the experiment. We used the variance components application of mixed linear models (Eq 2):

$$(Q_{ijkmn}-Q_{Mi})t^{-1}=\mu +S_{i}+\theta_{j}+T_{k}+W_{m}+S_{i}\theta_{j}+S_{i}T_{k}+S_{i}W_{m}+\theta_{j}T_{k}+\theta_{j}W_{m}+T_{k}W_{m}+e_{ijkmn}$$ (2)

where *Q*_*ijkmn*_ is the measured compound content at the end of the experiment (μg g^-1^ dry mass) in the soil type i, moisture level j, temperature k, worm treatment m, and replicate n. *Q*_*Mi*_ is the mean compound content at the beginning of the experiment (μg g^-1^) for the soil type i measured from parallel samples and t is the duration of the experiment (d, days). The calculated change rate (the right-hand side of the equation) for each treatment is directly comparable to *γ*_*ijkm*_ in Eq 1. A positive change rate refers to an increasing compound pool size. The application of this method allows evaluation of the significance of the treatment effects and their interactions.

We studied the temperature sensitivity of the C and N processes by assessing the release rates (Eqs 1 and 2) obtained in different temperatures. In cases where the response was positive or negative throughout the studied temperature range, we fitted a Q_10_ function into the dataset (Eq 3) using a non-linear least-square algorithm in Python [47].

$$k_{T}=k_{ref}Q_{10}^{\left(\frac{T-T_{ref}}{10}\right)}$$ (3)

Where *k*_*T*_ is the measured release rate at incubation temperature *T*, *k*_*ref*_ is the measured release rate at reference temperature *T*_*ref*_ (here 10°C), and *Q*_*10*_ is a fitted temperature sensitivity parameter. Q_10_ function could not be used in cases where the change rate turned from positive to negative or from negative to positive within the temperature range as it does not allow crossing the x-axis. The implications of this will be discussed later.

### Research permits

The study areas were located on state-owned land allocated to research and managed by Metsähallitus. Research activities in these areas are regulated in Partnership Agreement (2200/12 01 01 05 01/2018) between Metsähallitus and Natural Resources Institute Finland (Luke). The agreement allows the field activities done in this study including measurement of trees and soil sampling. No endangered or protected species were threatened in the field work.

## Results

### The effect of enchytraeid worms

**Fig 2:**
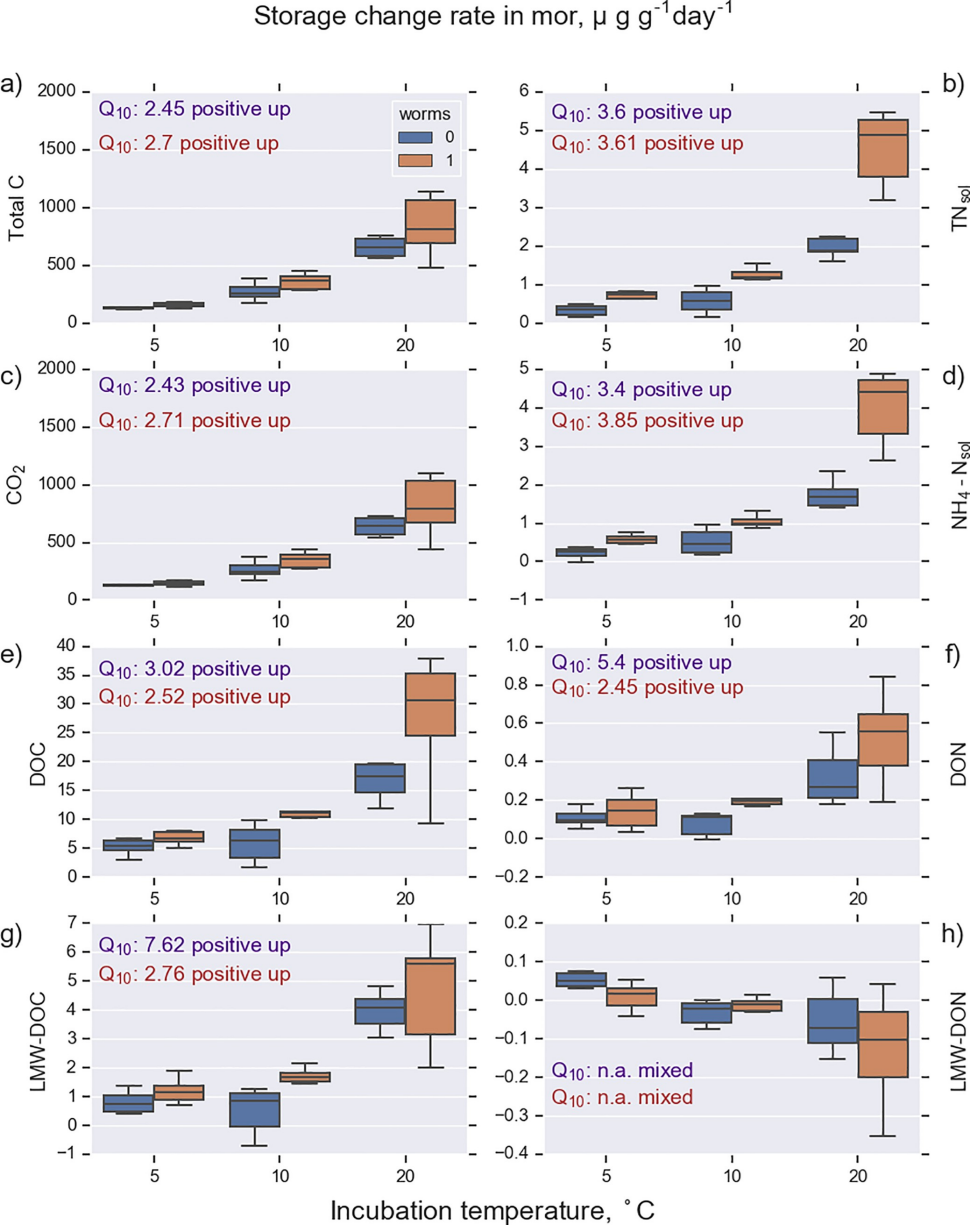
Release rate of CO_2_ and rates of change of C and N storage in soil solution collected from mor samples. Samples (n = 36) were incubated under constant ambient temperature of 5, 10, and 20°C with (worms 1) and without enchytraeid worms (worms 0). Q_10_ values (Eq 3) and the temperature response patterns (see Fig 6) are presented for each compound. The observed compounds were total carbon (Total C), total nitrogen (TN_sol_), carbon dioxide (CO_2_), ammonium nitrogen (NH_4_-N_sol_), dissolved organic carbon (DOC), dissolved organic nitrogen (DON), low-molecular-weight DOC (LMW-DOC), and low-molecular-weight DON (LMW-DON).

**Fig 3:**
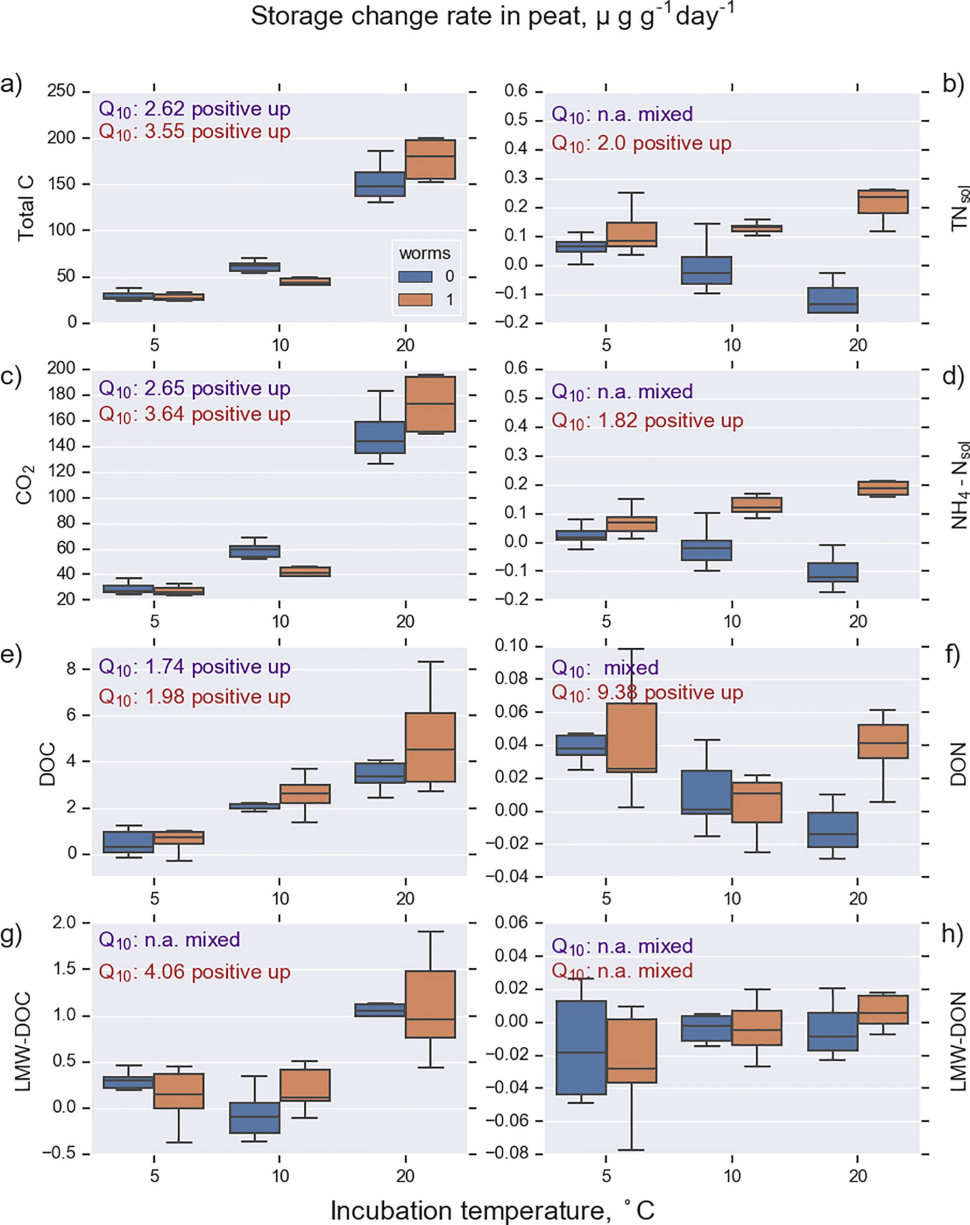
Release rate of CO_2_ and rates of change of C and N storage in soil solution collected from peat samples (n = 36). See Table 1 for abbreviations.

Enchytraeid worms increased the release rate of mineral N in both soil types and the effect was pronounced in the highest incubation temperature (Table 3, Figs 2 and 3). Worms enhanced CO_2_ efflux especially at high temperatures and moisture levels. The effect of enchytraeid worms on CO_2_ efflux was greater in mor than in peat (Table 3).

**Table 3:** Change rate (μg g^-1^ day^-1^) in dissolved N and C pools and CO_2_ release during the incubation calculated using mixed linear effect models (Eq 1). Parameter γ represents the change rate for mor (S_m_), no worms (W_0_), lower moisture status (θ_1_) at 5°C temperature (T_5_). The other treatment effects and the interaction effects (from W_1_ to T_20_ & S_p_) are expressed as a difference with respect to γ.

	TN_sol_		NH_4_-N_sol_		DON		LMW-DON		CO_2_		DOC		LMW-DOC	
γ	0.227	.	0.149		0.104	*	0.021		146.443	**	7.066	**	0.936	***
W_1_	0.609	***	0.524	***	0.030		-0.001		20.196		5.594	*	0.389	
T_10_	0.295	.	0.298	.	-0.006		-0.062		178.479	**	-0.301		-0.107	
T_20_	2.028	***	1.767	***	0.188	**	-0.169	**	488.843	***	11.872	***	2.992	***
θ_2_	-0.120		-0.141		0.000		0.036		-51.576		4.696	.	-0.201	
S_p_	0.073		0.101		-0.061		-0.072		-67.472		-9.204	***	-0.452	.
W_1_ & T_10_	0.213		0.169		0.043		0.001		-9.047		-1.353		0.631	*
W_1_ & T_20_	1.164	***	1.074	***	0.010		0.013		156.617	**	1.461		0.246	
W_1_ & θ_2_	0.164		0.173		0.034		-0.057		119.176	**	3.699		0.136	
W_1_ & S_p_	-0.981	***	-0.865	***	-0.050		0.023		-129.177	**	-6.605	**	-0.640	**
T_10_ & θ_2_	0.061		0.068		-0.008		-0.021		-40.197		-6.912	*	-0.223	
T_20_ & θ_2_	0.174		0.231		0.002		-0.030		128.162	*	-6.017	*	0.312	
T_10_ & S_p_	-0.455	**	-0.414	*	-0.037		0.106	.	-134.731	*	6.134	*	-0.198	
T_20_ & S_p_	-2.718	***	-2.414	***	-0.221	***	0.220	***	-502.837	***	-6.074	*	-2.405	***

Significance codes: 0 < *** < 0.001 < ** < 0.01 < * < 0.05. < 0.1

### The effect of temperature

**Fig 4:**
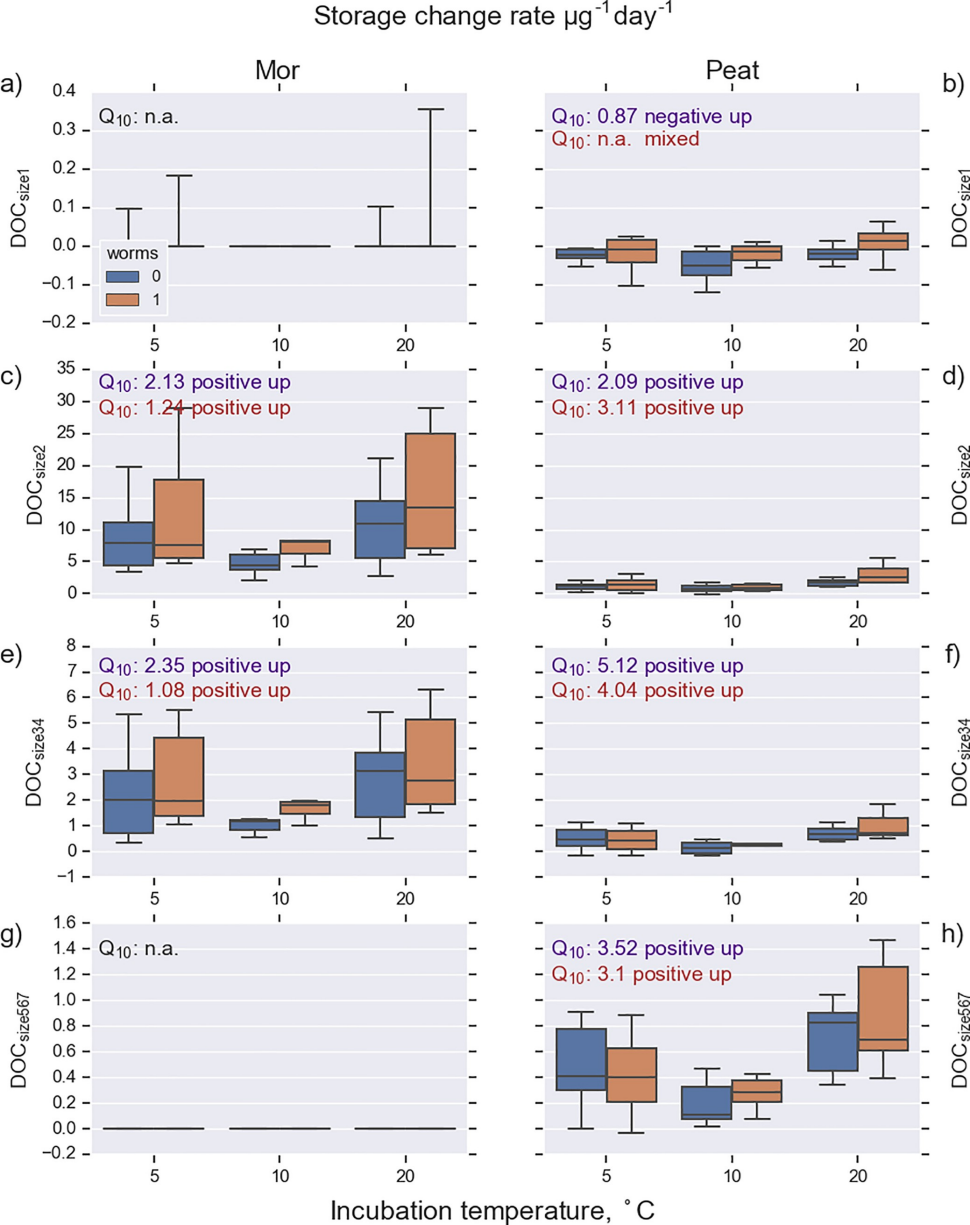
Rate of change in different dissolved organic carbon (DOC) molecular-size fractions. Molecular size decreases from DOC_size1_ to DOC_size7_. DOC_size1_ and DOC_size2_ belong to high-molecular weight-DOC (HMW-DOC); the remainder belong to low-molecular-weight DOC (LMW-DOC). See Table 1 for abbreviations.

Increasing temperature enhanced the release of CO_2_ and DOC both in mor and in peat. Furthermore, the release of TN_sol_, NH_4_-N_sol_, and DON increased with increasing temperature in mor. Interestingly, a contrasting pattern for N dynamics was observed for peat. In peat without worms, the amount of N in soil solution decreased; the decrease rate was most pronounced at the highest temperature. Examining the change rate in various molecular-size DOC pools revealed that the release of DOC in all size classes increased with increasing temperature (Fig 4, Table 4). However, this response was more pronounced in mor than in peat. The largest molecular-size fraction (DOC_size1_) and the smallest fraction (DOC_size5-7_) were practically absent in mor. Overall, the release of DOC in size classes 2–4 was smaller in peat than in mor. The destructive analyses showed that the microbial N and C decreased during the experiment and the decrease was most pronounced in the highest temperature and in the presence of worms (Table 5).

**Table 4:** Change rate (μg g^-1^ day^-1^) in different DOC molecular-size pools during the incubation (Eq 1). Parameter γ represents the change rate for mor (S_m_), no worms (W_0_), lower moisture status (θ_1_) at 5°C temperature (T_5_). The other treatment effects and the interaction effects (from W_1_ to T_20_ & S_p_) are expressed as a difference with respect to γ. Molecular size decreases from size class 1 to size class 7. DOC_size1_ and DOC_size2_ belong to HMW-DOC.

	DOC_size1_		DOC_size2_		DOC_size3-4_		DOC_size5-7_	
γ	0.005		3.922	***	0.421		0.164	
W_1_	-0.015		2.327	.	0.534		0.275	
T_10_	-0.001		1.587		0.340		0.251	
T_20_	0.019		10.279	***	2.736	***	1.501	***
θ_2_	-0.014		1.604		0.366		0.285	
S_p_	-0.073	**	-4.425	***	-1.077	**	-0.683	*
W_1_ & T_10_	0.023		-0.608		-0.021		-0.016	
W_1_ & T_20_	0.045		3.317	*	0.136		0.041	
W_1_ & θ_2_	0.021		1.995	.	0.605	.	0.367	
W_1_ & S_p_	0.004		-3.840	**	-0.800	*	-0.395	
T_10_ & θ_2_	-0.011		-2.024		-0.686		-0.465	
T_20_ & θ_2_	0.025		-2.123		-0.542		-0.390	
T_10_ & S_p_	0.032		0.964		0.620		0.424	
1T_20_ & S_p_	-0.008		-7.995	***	-1.143	**	-0.356	

Significance codes: 0 < *** < 0.001 < ** < 0.01 < * < 0.05. < 0.1

**Table 5:** Change rate (μg g^-1^ day^-1^) in microbial and extractable N and C pools during the incubation calculated using mixed random effect models (Eq 2). Parameter γ represents the change rate for mor (S_m_), no worms (W_0_), lower moisture status (θ_1_) at 5°C temperature (T_5_). The other treatment effects and the interaction effects (from W_1_ to T_20_ & S_p_) are expressed as a difference with respect to γ.

	NH_4_-N_ex_		ON_ex_		N_mic_		OC_ex_		C_mic_	
γ	1.7117	**	-2.4796	***	0.22122		-18.992	***	-28.6126	***
W_1_	1.4814	*	-0.07738		0.01171		0.23802		0.153	
T_10_	2.1706	**	-0.18972		-0.07804		0.23403		-1.9596	
T_20_	6.2275	***	-0.83243	***	-1.66107	***	1.2442		-6.3842	***
θ_2_	-0.7672		0.0745		0.28896		0.08451		1.0405	
S_p_	0.2616		1.22592	***	-0.78868	*	11.3633	***	-6.2476	***
W_1_ & T_10_	1.1671		-0.07764		-0.52194		0.75265		-1.0363	
W_1_ & T_20_	2.9988	***	-0.57102	***	-0.96085	*	0.22267		-2.9808	.
W_1_ & θ_2_	0.8784		-0.22541	.	-0.02266		-0.298		-1.0362	
W_1_ & S_p_	-2.1961	***	0.39969	**	0.26143		0.12227		1.1673	
T_10_ & θ_2_	0.7919		0.15578		-0.27923		2.0982		0.3388	
T_20_ & θ_2_	1.2233	.	0.03276		-0.80728	*	2.44092	.	-2.3267	
T_10_ & S_p_	-2.6848	***	0.2035		0.62903	.	-1.31926		2.7958	.
T_20_ & S_p_	-7.8357	***	1.24918	***	2.86576	***	-0.91912		8.8824	***
θ_2_ & S_p_	-0.3429		-0.02197		0.19131		-1.56022		0.3652	
R^2^	0.92		0.95		0.72		0.85		0.6	

Significance codes: 0 < *** < 0.001 < ** < 0.01 < * < 0.05. < 0.1

### The effect of soil moisture

**Fig 5:**
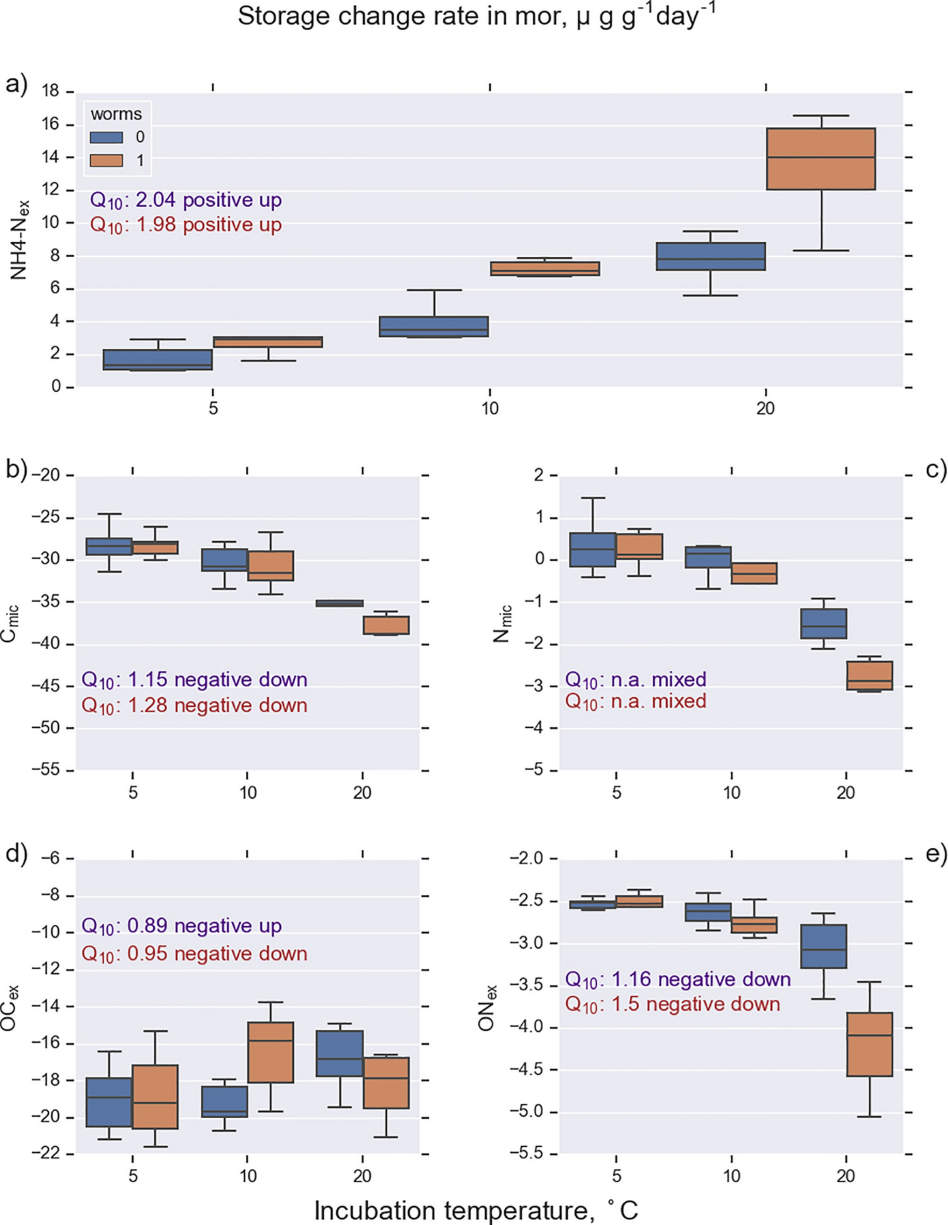
Rate of change in K_2_SO_4_-extractable C and N pools in mor samples (n = 36). Incubation was conducted at 5, 10, and 20°C with (worms 1) and without enchytraeid worms (worms 0). The measured pools were ammonium (NH_4_-N_ex_), microbial C, microbial N, total organic C (OC_ex_), and total organic N (ON_ex_). See Table 1 for abbreviations.

**Fig 6:**
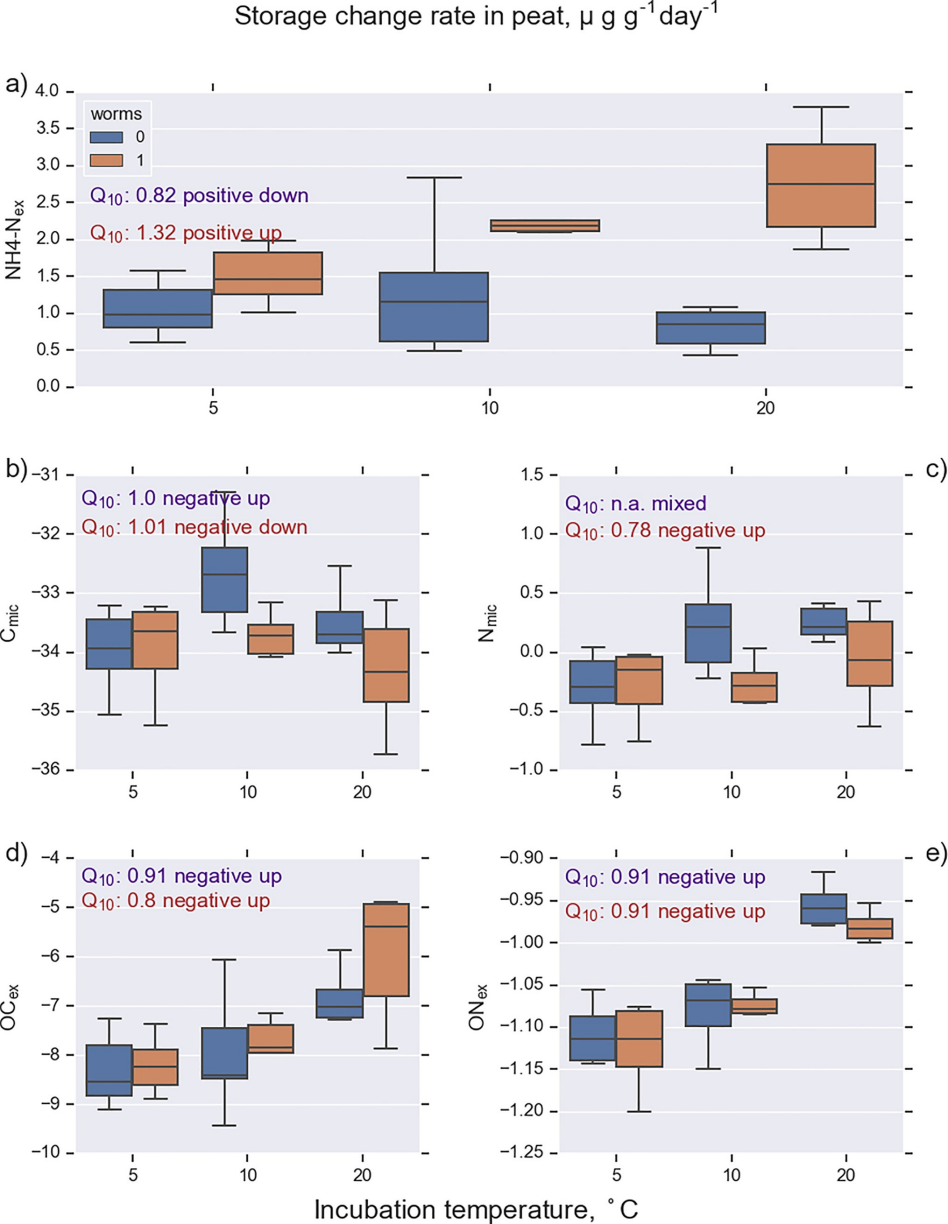
Rate of change in K_2_SO_4_-extractable C and N pools in peat samples (n = 36). Incubation was conducted at 5, 10, and 20°C with (worms 1) and without enchytraeid worms (worms 0). The measured pools were ammonium (NH_4_-N_ex_), microbial C, microbial N, total organic C (OC_ex_), and total organic N (ON_ex_). See Table 1 for abbreviations.

The moisture range in the study was rather small and therefore moisture alone did not have a significant effect on the C and N release. However, at the highest temperature, the larger water content increased CO_2_ release and decreased DOC release. As moisture played a minor role in the results, we combined the moisture levels (θ_1_ and θ_2_) for Figs 2–6.

### Dissolved and extractable N

The change rate of NH_4_-N_ex_ followed the pattern of NH_4_-N_sol_, but the absolute values of the NH_4_-N_ex_ were generally 2 to 10 times higher than for the dissolved fraction (Figs 5 and 6, Tables 3 and 5). An interesting pattern was exposed when comparing signs of the change rate components between extractable organic nitrogen (ON_ex_) and dissolved organic nitrogen (DON). In most cases, when ON_ex_ components were positive, the respective DON component was negative and vice versa (Tables 3 and 5), indicating exchange processes between the adsorbed and dissolved pools. There was also a similar inverse connection between the components of NH_4_-N and ON_ex_.

### Temperature sensitivity

The experiment revealed the following five different temperature sensitivity patterns: positive upwards (Q_10_ > 1), positive downwards (0 < Q_10_ < 1), negative upwards (0 < Q_10_ < 1), negative downwards (Q_10_ > 1), and mixed responses (Fig 6). A negative response was obtained for negative k_ref_ values in Eq 3. The data allowed us to describe 68 temperature response curves (Figs 2–6). A positive upwards pattern was the most common and occurred in 37 cases (Table 6). This pattern was found for CO_2_ and DOC in all treatments, NH_4_-N_sol_ and DON in mor, and NH_4_-N_sol_ in peat where worms were present. The second most common was the mixed response (16 cases), where the sign of the response changed from positive to negative or from negative to positive with increasing temperature. It is noteworthy that the Q_10_ function cannot be applied for the mixed type. A negative response, where the release rate was always negative, was typically found for destructive analyses (NH_4_-N_ex_ in peat without worms, C_mic_ and ON_ex_ in mor).

**Table 6:** Occurrence of different temperature response patterns in the multifactorial laboratory experiment. The response shapes and Q_10_ values are presented in Figs 2–6.

	Dissolved		HPLC		Destructive		
	mor	peat	mor	peat	mor	peat	SUM
Positive upwards (Q_10_ > 1)	14	10	4	6	2	1	37
Positive downwards (0 < Q_10_ < 1)				1		1	2
Negative downwards (Q_10_ > 1)					4	2	6
Negative upwards (0 < Q_10_ < 1)					2	5	7
Mixed response	2	6	4	1	2	1	16
Total							68

C release in mor was considerably higher when worms were present (Fig 2). DOC release drastically increased when temperature rose from 10°C to 20°C; at the highest temperature the worms had the largest effect on DOC release. Enchytraeids increased the temperature sensitivity of C release in peat. The temperature sensitivity of total N release was higher than that of total carbon in mor. The difference in C release with and without worms emerged with increasing temperature. The new finding in our study was that mineral N release in peat was totally dependent on worms; there was no net release of N in samples without worms. On the contrary, N keeps accumulating in samples in the absence of worms. Thus, a negative temperature sensitivity emerged; immobilization increased with increasing temperature.

## Discussion

### Temperature sensitivity patterns

**Fig 7:**
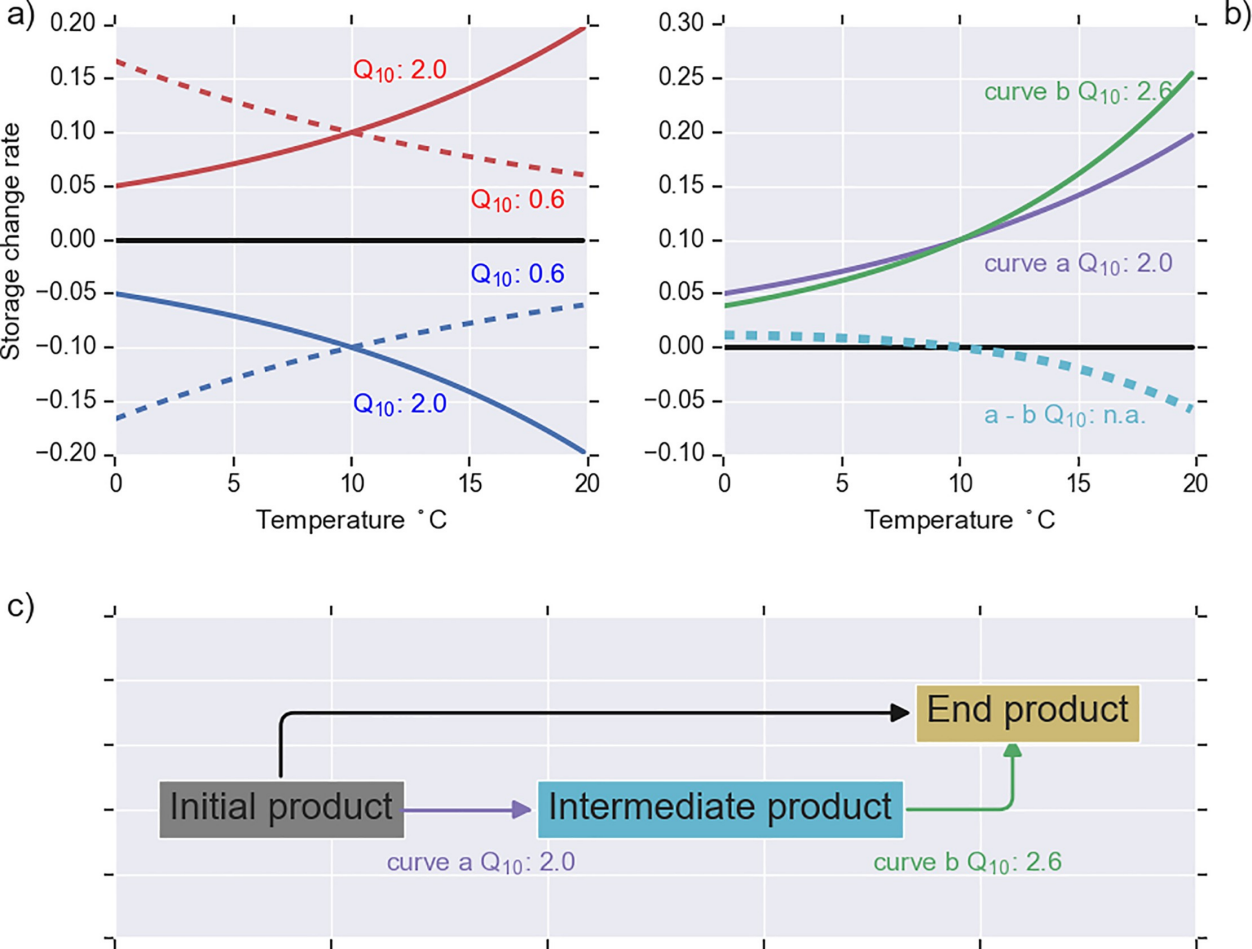
Schematic presentation of different temperature sensitivity patterns. In panel a) the solid red line refers to the positive upwards pattern (k_ref_ > 0 and Q_10_ > 1; Eq 3), the dashed red line refers to the positive downwards pattern (k_ref_ > 0 and 0 < Q_10_ < 1), the solid blue line is the negative downwards pattern (k_ref_ < 0, Q_10_ > 1), and the dashed blue line is the negative upwards pattern (k_ref_ < 0, 0 < Q_10_ < 1). The mixed temperature response (panel b, dashed light blue line) occurs for change rates typical for intermediate decomposition products (panel c) when the temperature sensitivity of the incoming and outgoing fluxes are different (panels b and c).

This study presents a unique, multifactorial experiment consisting of a broad set of abiotic and biotic factors that control the temperature sensitivity of organic C and N decomposition processes of boreal organic soils. Traditionally, incubation studies use small volumes of sieved soil. In contrast, we used large, intact soil cores that are more likely to reflect processes in field conditions. There are only few studies where temperature sensitivity of N [9,17,18] or DOC processes [48] have been studied; to our knowledge this is the first study where temperature sensitivity of different molecular size fractions of DOC and DON have been quantified. The wide range of the studied C and N compounds (altogether 17 compounds in two soils and two worm treatments equaling 68 different combinations), including intermediate and end products of decomposition, allowed us to recognize different temperature sensitivity patterns (Fig 7A and 7B). The most common pattern (37 out of 68) was a positive temperature response where the storage change rate was positive throughout the studied temperature range and where the rate increased with increasing temperature (Table 6, Fig 7A, solid red line). This pattern was observed for CO_2_ and DOC release (Figs 2 and 3); Q_10_ values ranged from 1.7 to 3.6. This falls within the range reported for temperate, boreal, and arctic organic soils [12,49,50].

ON_ex_ and C_mic_ (e.g. Fig 5B and 5E) represented a negative temperature response pattern where the storage change rate was negative throughout the studied temperature range and decreased with increasing temperature (Fig 7A, solid blue line). This emerges because higher temperature is likely to promote the biodegradation of ON_ex_ into NH_4_-N and thereby decrease the storage of ON_ex_. The Q_10_ value also has a meaningful interpretation in these cases; the negative sign is only a matter of perspective, since a decrease in one storage is an increase in another.

Sixteen out of 68 studied temperature sensitivity patterns represented the mixed type (Fig 7B, dotted light blue line). In these cases, the sign of the storage change rate flipped from negative to positive or vice versa within the studied temperature range. Q_10_ function is not applicable in these cases as it does not allow changing the sign. The mixed pattern was typically connected to intermediate decomposition products, where input and output fluxes with different temperature sensitivities may change the storage (Fig 7B and 7C). The mixed type was especially typical for N processes. This highlights the complex nature of N temperature response and may clarify results from several other studies that have reported variable or unclear temperature responses for N mineralization [13,14,17,18,51].

### Temperature sensitivity patterns in context of kinetic theory

Conant et al. [52] identified three sets of processes that potentially affect the temperature sensitivity of decomposition: depolymerization of biochemically complex compounds; changes in microbial enzyme production while acclimating to warmer temperature; and processes that limit the availability of substrates for the decomposition process (e.g. adsorption and desorption).

Temperature sensitivity of decomposition and biodegradation processes can be described through kinetic theory as reviewed by Davidson and Janssens [53]. The basis of the theory can be expressed through temperature-driven Arrhenius kinetics combined with Michaelis-Menten enzyme kinetics. Arrhenius kinetics describes the relative reaction rate as affected by temperature, whereas Michaelis-Menten kinetics describes the reaction rate as affected by enzyme substrate availability. When the maximum reaction rate (*V*_*max*_) and Michaelis-Menten constant *K*_*m*_ (representing the substrate concentration at which the reaction rate is half of *V*_*max*_) have their own temperature sensitivities [53], the Michaelis-Menten kinetics can be rewritten in the form

$$k=\frac{V_{max}Q_{10,V_{max}}^{\frac{T-T_{ref}}{10}}(S)}{K_{m}Q_{10,K_{m}}^{(T-T_{ref})/10}+(S)},$$ (4)

where *Q*_10,*Vmax*_ and *Q*_10,*Km*_ are the temperature sensitivities of *V*_*max*_ and *K*_*m*_, respectively, and (S) is substrate concentration. Now, when the concentration exceeds *K*_*m*_, the overall temperature sensitivity is governed by the temperature sensitivity of *V*_*max*_. If, on the other hand, the substrate concentration is limiting the reaction, the overall temperature sensitivity reflects the combined effect of the temperature sensitivities of both *V*_*max*_ and *K*_*m*_. It is notable that in the situation where the substrate is limiting the reaction, the affinity of enzyme to substrate decreases with increasing temperature and reaction slows down, even though *V*_*max*_ increases. This provides an explanation for Q_10_ < 1 (Fig 7A, dotted red and blue line) and results in positive downwards and negative upwards temperature sensitivity patterns.

The complex temperature sensitivity patterns in N processes could be partly explained by enzyme chemistry. The activities of some N-acquiring enzymes have been found to increase strongly with temperature (protease, urease), while others (amidase) have been found to be insensitive to temperature and controlled instead by C availability [54]. Especially in N-poor peat, amidase activity can be detected without a labile C source probably because microbes are N limited [26]. Furthermore, the temperature sensitivity of some C-acquiring enzymes (betaglucosidase) have been found to respond differently to pH than N-acquiring enzymes (N-Acetyl-Glucosaminidase; NAGase decomposing chitin). Consequently, N release can be either less or more temperature sensitive than C release depending on pH [55], which may partly explain the different responses of mor and peat in our study and the variable responses also in previous studies.

### Role of enchytraeid worms

Enchytraeids overall enhanced the release of N and CO_2_. The effect of enchytraeid worms on CO_2_ efflux was greater in mor than in peat (Table 3). DOC release was also enhanced by the enchytraeids (Tables 3 and 4) and was clearest at the highest temperature (Fig 2E). The enhanced mineralization and release of dissolved organic compounds is consistent with earlier findings [5,27,56]. Enchytraeids played a crucial role in N cycling especially in peat. In peat without worms, the N amount in soil solution decreased; the decrease rate was most pronounced at the highest temperature. This emphasizes the role of soil fauna in the nutrient release of N-poor peat soils. Enchytraeids can stimulate decomposition by processing organic matter into a more labile form [57] or by excreting N-rich compounds [58]. In the absence of worms, during the incubation N appears to be immobilized in peat, either by adsorption on soil particles or by assimilation into microbial biomass. The presence of enchytraeids increased the temperature sensitivity of C release. To our knowledge, the effect of enchytraeids on the temperature sensitivity of C and N release has not been previously reported.

Microbial N and C decreased during the experiment in mor and the decrease was most pronounced at the highest temperature (reflecting the higher loss of labile C at a higher incubation temperature [49]) and in the presence of worms (reflecting the higher grazing pressure on microbial biomass). The concomitant change in C availability and quality (loss of labile C) and microbial community (decrease in biomass and decrease especially in fungal biomass) has been previously shown in incubation studies [12].

Enchytraeids are common in forest soils and their population may fluctuate according to season [22] and due to different disturbances, such as forest management practices [59]. In this experiment we compared the presence and absence of enchytraeids. Even though the absence of enchytraeids in field conditions is unlikely, our results provide a useful estimate for the maximum range for how the fluctuating enchytraeid population may reflect C and N processes at the ecosystem level as has been demonstrated by Lappalainen et al. [5].

### Mor and peat

The soluble storage change rates for C and N were almost an order of magnitude higher in mor than in peat (Figs 2 and 3), although the microbial biomass at the beginning of the experiment was almost equal in both soil types (Table 2). However, the CN ratio in the microbial biomass was drastically different (8.8 for mor and 22.8 for peat). This may reflect a higher N limitation in peat than in mor, which may slow down the biological activity in soil. The CN ratio of the decomposing material and the Q_10_ values for peat were higher than for mor (Figs 2A and 3A), which is consistent with the CQT hypothesis [7]. However, the N release in peat responded less to increasing temperature in peat than in mor. DOC release increases with temperature, and smallest and largest molecule size was missing from mor, which is consistent with results shown by Lappalainen et al. [5].

### Implications to global warming

Q_10_ is a robust and computationally efficient approach that enables upscaling experimental decomposition studies into the ecosystem level and beyond to a global scale. Q_10_ applies easily available input, such as vegetation type and temperature scenarios, making it a suitable approach e.g. for regional, spatially explicit modeling studies. In this study, we observed higher temperature sensitivity for peat compared to mor, suggesting that peatlands are more vulnerable to climate warming than mor-covered upland soils. From a C balance point of view, the primary production of vegetation is, however, equally important and largely depends on the N mineralization from decomposition of organic material [6,10]. Our results indicate that in peatlands much less N is released per unit mass of released C. In mor, however, the temperature sensitivity for N release was higher than C release (Fig 2A and 2B). Consequently, it is likely that primary production in peatlands does not increase to the same extent as in upland sites.

From a modeling point of view, the feedback loop from decomposition through N release to primary production of vegetation is essential. As pointed out by Novem Auyeueng et al. [18], ecosystem models that assume that warming will consistently increase N mineralization rates and inputs of plant-available N may overestimate the increase in terrestrial productivity and the magnitude of an important negative feedback to climate change. However, Q_10_ can be made more dynamic when we account for the dependency of temperature sensitivity of N on environmental factors such as rainfall and edaphic conditions [10]. There are limitations to the Q_10_ approach; thus more mechanistic models, which can account for kinetic theory, soil pH, substrate characteristics, and soil physical conditions such as hydrology and oxygen supply are needed to increase process-based understanding. However, such models tend to be computationally demanding and require input that is not available from large areas.

## Supporting information

S1 Dataset Original data. (XLSX)
